# Cell therapy for neuropathic pain

**DOI:** 10.3389/fnmol.2023.1119223

**Published:** 2023-02-27

**Authors:** QingHua Yin, TianHao Zou, ShuJun Sun, Dong Yang

**Affiliations:** ^1^Department of Pain, Union Hospital, Tongji Medical College, Huazhong University of Science and Technology, Wuhan, China; ^2^Institute of Anesthesia and Critical Care Medicine, Union Hospital, Tongji Medical College, Huazhong University of Science and Technology, Wuhan, China

**Keywords:** neuropathic pain, cell therapy, transplantation, stem cell, analgesia, nerve injury

## Abstract

Neuropathic pain (NP) is caused by a lesion or a condition that affects the somatosensory system. Pathophysiologically, NP can be ascribed to peripheral and central sensitization, implicating a wide range of molecular pathways. Current pharmacological and non-pharmacological approaches are not very efficacious, with over half of NP patients failing to attain adequate pain relief. So far, pharmacological and surgical treatments have focused primarily on symptomatic relief by modulating pain transduction and transmission, without treating the underlying pathophysiology. Currently, researchers are trying to use cell therapy as a therapeutic alternative for the treatment of NP. In fact, mounting pre-clinical and clinical studies showed that the cell transplantation-based therapy for NP yielded some encouraging results. In this review, we summarized the use of cell grafts for the treatment of NP caused by nerve injury, synthesized the latest advances and adverse effects, discussed the possible mechanisms to inform pain physicians and neurologists who are endeavoring to develop cell transplant-based therapies for NP and put them into clinical practice.

## 1. Introduction

Neuropathic pain (NP) is defined by The International Association for the Study of Pain (IASP) as “pain caused by a lesion or disease of the somatosensory system” (Scholz et al., 2019). The prevalence of NP may stand somewhere at 7–8% in the general public and may be up to 20–25% in patients with chronic pain (Bouhassira, 2019). NP can originate from peripheral or central nerves, and is seen in common diseases, such as peripheral or central nerve injury, trigeminal neuralgia, postherpetic neuralgia, diabetic peripheral neuropathy, and multiple sclerosis. The symptoms associated with NP present as spontaneous pain, hyperalgesia and allodynia, and are usually described in terms of relatively typical pain qualities, such as burning pain, shooting pain, electric shock-like pain, pins-and-needles pain, dysesthesia and brush allodynia, etc (Bouhassira, 2019; Finnerup et al., 2021). In addition, NP exerts a tremendous influence on patients’ sleep, life quality, and causes anxiety and depressive symptoms, and poses great burden on healthcare use. Current treatment for NP consists mainly of pharmacotherapies, with moderate-to-high quality evidence supporting the use of calcium channel action modulators (pregabalin, gabapentin), 5-hydroxytryptamine-norepinephrine reuptake inhibitors (venlafaxine, duloxetine) and tricyclic antidepressants (amitriptyline, nortriptyline). In fact, they are used as the first-line therapeutic regimes for NP (Gierthmuhlen and Baron, 2016; Macone and Otis, 2018). Nonetheless, since the analgesic effect of existing drugs is limited and dose-dependent side effects, their therapeutic effectiveness is far from ideal, and over 50% patients with NP do not attain sufficient pain relief (Basbaum and Braz, 2016). Therefore, new treatment strategies with durable effects are urgently warranted.

Pathophysiologically, peripheral and central sensitization is responsible for NP. Cellular maladaptive structural changes, cell-to-cell interactions, and molecular signaling are pivotal players in the sensitization following nociceptive stimuli (Finnerup et al., 2021). Moreover, a series of changes, such as ion channel alternation, activation of immune cells, glial cell-derived mediators, and epigenetic regulation are also implicated in the process of neural sensitization. In recent years, research effort has been directed on the biochemical and molecular alterations that contribute to peripheral and central sensitization, but few newly identified targets have been translated into clinical application (Basbaum and Braz, 2016). Since NP is mechanistically complicated, its treatment remains a challenge for clinicians. Therefore, to improve the efficacy of NP therapy it is of great significance to understand its underlying mechanisms and to work out treatments that target the mechanisms.

Cell therapy is a potentially effective approach to relieve nerve injury and NP (Huang et al., 2012; Chen et al., 2013; Assinck et al., 2017). Prior studies have shown that cell-based therapeutic modalities are effective against various common chronic pain syndromes, such as discogenic pain, NP, osteoarthritis, musculoskeletal disorders (meniscal lesions, femoral head necrosis), among others (Chakravarthy et al., 2017). Neurorestoratologically, possible neurorestorative mechanisms of cell transplantation might involve neuroprotection, neurotrophy, neuroreparation, neuroregeneration, neuromodulation, or neuroconstruction, as well as immunomodulation and enhancement of the microcirculation.

In this review study, the studies regarding cell therapy for the management of NP induced by nerve injury over the past 10 years were retrieved from Pubmed, Cochrane, and CBM. We synthesized the studies, categorized the types of cellular grafts, discussed the latest advances, adverse effects, and possible mechanisms of the NP therapies, to provide information for pain physicians and neurologists who are endeavoring to develop cell therapy for the treatment of NP.

## 2. Types of cell grafts

### 2.1. Neural stem cells

Neural stem cells (NSCs) are a class of pluripotent cells with division potential, self-renewal ability, and capability of differentiating into neurons, astrocytes and oligodendrocytes. NSCs are ubiquitously present in a wide array of tissues, including the cerebral cortex, olfactory bulb, hippocampus, striatum, lateral ventricle ependymal/subventricular zones, and the spinal marrow of embryos, fetuses, and adults (Tuazon et al., 2019).

Initially, the notion of using cell transplantation for the treatment of NP was premised on the fact that stem cells act as a kind of totipotent cells that can replace damaged nerve cells and deliver trophic factors to the site of the lesion. NSCs make a superior candidate cell type for cell therapy since they are highly capable of differentiating into neurons and glial (Franchi et al., 2014).

Previous studies have shown that NSCs can be induced to differentiate into stem cells and promote axonal regeneration. Wang et al. (2017) retrospectively reviewed the role of NSCs in the repair of peripheral nerve injury, and found that, NCSs not only possessed nerve regenerative and neuroprotective effects, but also secreted a variety of factors to enhance the survival of motor and sensory neurons and to promote angiogenesis. Further experiments confirmed that neural stem cells could interact bidirectionally with resident cells in the damaged microenvironment. Du et al. (2019) locally injected NSCs into the injury site of the spinal cord injury (SCI) rat model, and the transplanted NSCs could regulate the expression of P2X receptor and improve the microenvironment after SCI, thereby enhancing neural regeneration and functional movement. A study treated patients with chronic SCI damage by multiple intramedullary injections of human-derived CNS stem cells and found that, after transplantation, an interaction took place between stem cells and the microenvironment (Levi et al., 2018).

In addition to relieving central NP, NSCs transplantation could also effectively ease NP caused by peripheral nerve injury. In a rat sciatic nerve transection (SNT) model, neural crest stem cells attenuated NP and improved motor function (Zhang et al., 2021). Lee et al. (2016) found that NSCs could relieve NP and promote nerve regeneration in a rat model, and a small amount of persistently expressed vascular endothelial growth factor (VEGF) could effectively modulate nerve function. However, uncontrolled overexpression of VEGF might pose the risk of causing tumor formation.

Currently, a great many animal studies confirmed that NSCs provide effective relief of NP. Prior studies showed that NSCs appeared to be more effective for the peripheral NP than for the central one, but the relevant literature is scanty and further comparative studies are needed.

A small number of clinical trials employed human-derived neural stem cells for treating NP due to chronic SCI (Curtis et al., 2018), and their efficacy and safety were experimentally supported, but the sample size was small, and more trials are warranted to support their clinical application.

### 2.2. Olfactory ensheathing cells

Glial cells that surround and enclose the olfactory nerve bundle and the outer layers of the olfactory bulb, are collectively known as olfactory ensheathing cells (OECs). They possess the unique ability to transgress the peripheral nervous system (PNS) and central nervous system (CNS) environments (Andrews et al., 2016). And OECs have also been shown to have neuro-regenerative functions (Zhang et al., 2020a). Because of this property, olfactory ensheathing cells are also seen as a good choice for treating nerve injury and NP.

Zhang et al. (2019a,b, 2020a,e) in a series of studies, used OECs transplantation for treating NP in chronic constrictive injury (CCI) model rats, and confirmed that OECs transplantation could promote motor recovery and mitigate pain. Their studies found that OECs secreted various neurotrophic factors (e.g., neurotrophic Y, neurotrophic factor 3, enkephalin, and endorphin), inhibited the formation of colloid scar and cavities, and facilitated regeneration and myelination of new axons, and they were effective for the treatment of NP. At the same time, they also found that the inhibition of the P2 purinoceptor family was an important part of the process. Further studies have revealed that OECs transplantation inhibit P2X2 receptor (Zhao et al., 2015; Zhang et al., 2020c), P2X4 receptor (Zheng et al., 2017; Zhang et al., 2019b, 2020b), and P2X7 receptor (Zhang et al., 2019a, 2020a) overexpression mediated NP. Wu et al. (2011) found that delayed OECs transplantation also can effectively inhibit hyperalgesia and allodynia after dorsal root injury.

A meta-analysis published in 2018 (Nakhjavan-Shahraki et al., 2018) reviewed 40 studies related to the effect of OEC transplantation on NP and functional recovery following spinal cord damage, and concluded that OEC transplantation significantly improved post-injury motor function, but had no effect on allodynia and might even lead to a relative exacerbation of nociceptive hyperalgesia, especially 8 weeks after OECs transplantation. Animal experiments by Lang et al. (2013) found that transplantation of olfactory bulb OECs into the rat hemispheric spinal cord caused hyperalgesia, and they observed the activation of ERK and the up-regulation of the brain derived neurotrophic factor (BDNF) following OECs transplantation, which may be related to hyperalgesia. BDNF is an important regulator of pain, and studies have shown that it exerts both anti-nociceptive and anti-inflammatory actions and pro-nociceptive effects (Cao et al., 2020). The BDNF/tyrosine receptor kinase B (TrkB) signaling pathway is closely related to NP. BDNF polymorphisms can interfere with BDNF role in pain perception, resulting in pain relief or exacerbation, and existing researches suggested that genetic factors such as Val66Met are involved (Cappoli et al., 2020). The authors consider that this may be related to the controversial result of relieving NP after OECs transplantation, but no specific studies on this pathway and BDNF genotypes after transplantation have been reported.

However, two studies (Feron et al., 2005; Tabakow et al., 2013) on autologous OECs in the treatment of human SCI failed to observe any hyperalgesia or serious adverse effects. A study by Mackay-Sim et al. (2008) performed OECs transplantation in patients with traumatic injuries to the thoracic spine and observed no adverse effects during 3-year follow-up. These experiments have confirmed the feasibility of olfactory sheath cell transplantation to some extent, but further validation is needed. In order to further address the risk of exacerbated hyperalgesia upon OECs transplantation, some studies used combined transplantation to treat NP. Co-transplantation of NSCs and OECs not only promoted the survival of NSCs, but also reversed the hyperalgesia caused by OECs (Luo et al., 2013).

Due to their source abundance, easy availability, and high viability in *ex vivo* culture, OECs have good prospect of being used for treating NP in clinical practice. However, experiments with longer observation time are still needed to verify their long-term effects and safety.

### 2.3. Mesenchymal stem cells

As a kind of adult stem cells, mesenchymal stem cells (MSCs) can be obtained from various sources, such as bone marrow, umbilical cord, adipose tissue and placenta, and can be induced to differentiate into endoderm, mesoderm, and ectoderm cell lines. Mesenchymal stem cells are abundant in source and easy to expand, do not express the major histocompatibility complex class II cell surface receptor HLA-DR, and are characterized by low immunogenicity, high immunomodulation and good phenotypic stability. As a result, they can be used for the treatment of diseases involving neuroinflammatory components, such as immune diseases, NP, etc (Franchi et al., 2014; Jwa et al., 2020) A great number of animal experiments have demonstrated that MSCs have great potential to be used for alleviating NP symptoms, such as allodynia and hyperalgesia. Moreover, most of the trials did not observe MSCs-related adverse reactions (Wang et al., 2020). MSCs can be administered intravenously, intrathecally, and topically at the site of injury, and can be transported and collected at the site of injury regardless of the route of administration, a phenomenon known as post-transplantation homing (Chakravarthy et al., 2017; Han et al., 2019; Xie et al., 2022).

#### 2.3.1. Bone marrow mesenchymal stem cells

Bone marrow mesenchymal stem cells (BM-MSCs), the most representative type of MSCs, are currently widely used in the treatment of experimental NP. A systematic review (Wang et al., 2020) published in 2020 included 17 studies on the transplantation of MSCs in animal models of NP elicited by perineural injury. Of the studies, 14 used MSCs derived from bone marrow. The mechanisms by which BM-MSCs improve NP and facilitate motor recovery included anti-inflammatory regulation, inhibition of phenotypic activation of microglia and astrocytes and improvement of synaptic transmission, and promotion of neuronal network repair (Yamazaki et al., 2021). BM-MSCs are effective against NP in both peripheral and central models of neurogenic animals, but appear to be more effective for NP induced by peripheral approaches. A meta-analysis by Hosseini’s team (Hosseini et al., 2015) further found that BM-MSCs transplantation improved allodynia and exerted no influence on hyperalgesia with the exception of cell transplantation performed in the first 4 days after injury. Schäfer et al. (2014) found that intrathecal injection of BM-MSCs did not have any beneficial impacts on nociceptive pain, such as allodynia and hyperalgesia in female mice with partial sciatic nerve ligation (PSNL). Gender difference in NP is gaining attention and studies (Schäfer et al., 2014; Hsu et al., 2020) showed that BM-MSCs appeared to have no beneficial effect on NP in female mice. Researchers have suggested that this might be ascribed to gender-dependent driven neuroinflammation, with the sex dimorphism involved in microglial activation (Coraggio et al., 2018), but further subgroup analyses are needed for verification.

#### 2.3.2. Adipose tissue-derived mesenchymal stem cells

Adipose tissue-derived mesenchymal stem cells (AD-MSCs) are another kind of cells typical of MSCs, and their greatest advantage lies in that they can be obtained in large quantities from mature subcutaneous adipose tissue by using a low-invasive procedure. AD-MSCs exert analgesic and neuroprotective effects through their anti-inflammatory actions, mainly by regulating pro-inflammatory or anti-inflammatory cytokines and neurotrophic factors (Miyano et al., 2022). However, it is worth noting that AD-MSC grafts do not relieve all types of NP. Jwa et al. (2020) discovered that a single intrathecal injection of AD-MSCs could significantly reduce cold allodynia induced by L5 spinal nerve ligation, but no significant improvement was observed in mechanical allodynia. Furthermore, repeated intrathecal injections of AD-MSCs selectively reduced cold allodynia.

#### 2.3.3. Umbilical cord mesenchymal stem cells

Umbilical cord mesenchymal stem cells (UC-MSCs) can be collected non-invasively by autologous or allogeneic donors, and have a higher expansion capacity than BM-MSCs. Meanwhile, UC-MSCs have strong proliferation ability, low bacterial/viral infection rate, low immunogenicity and good immunosuppressive ability (Franchi et al., 2014; Joshi et al., 2021). The analgesia induced by UC-MSCs is achieved through their anti-inflammatory and remyelination actions, and umbilical cord-derived cells have a tendency to express genes involved in angiogenesis and intracellular matrix renewal, which are conducive to the repair of injured spinal cord tissue (Wu et al., 2020). Yousefifard et al. (2016) used UC-MSCs and BM-MSCs for the treatment of NP in an SCI animal model, and both could promote functional recovery and ease allodynia and nociceptive hyperalgesia, but UC-MSCs outperformed BM-MSCs in terms of both survival and electrophysiological performance at 8 weeks, suggesting that UC-MSCs are a candidate of choice in terms of neurological repair.

#### 2.3.4. Mesenchymal stem cell exosomes

Moreover, some researchers discovered that MSC exosomes also contribute to pain relief, and tried to use them for cell-free treatment of NP. Exosomes are extracellular vesicles of 30–160 nm secreted by endosomal membranes, containing proteins, lipids, and nucleic acids, and are transported to proximal and distant targets through the circulation, to participate in intercellular communication and alter protein expression in target cells (Jean-Toussaint et al., 2020). The particles smaller than 200 nm are defined as small extracellular vesicles (sEV) by the International Society for Extracellular Vesicles, rather than being taken as exosomes. Shiue et al. (2019) intrathecally injected UC-MSCs exosomes into rats with spinal nerve ligated and found that a single intrathecal injection reversed hyperalgesia, and continuous intrathecal administration suppressed the overexpression of c-Fos, CNPase, GFAP and Iba1 in the spinal cord and DRG, achieving good preventive and reversal effects on NP induced by nerve ligation. Moreover, immunofluorescence assay revealed that UCMSC exosomes had homing ability. Hsu et al. (2020) also found that MSCs-derived exosomes had immunotherapeutic activities comparable to MSCs *per se*, possessed anti-nociceptive, anti-inflammatory and neurotrophic effects, and promise to become a treatment modality for NP. Nevertheless, given that current protocol for isolating and purifying exosomes has yet to be refined (D’Agnelli et al., 2020), the preparation procedures are labor-intensive and costly, and current research is still limited to animal experiments, the treatment strategy is still a long way from its clinical application.

### 2.4. Bone marrow mononuclear cells

Bone marrow mononuclear cells (BMMCs), a mixed population of cells, include monocytes, lymphocytes, hematopoietic stem cells, and endothelial cell precursors. BMMCs can be isolated from bone marrow by density gradient centrifugation and have been shown to be able to relieve experimental NP. Takamura et al. (2020) found that intrathecal injection of BMMCs attenuated NP in a mice model of spinal nerve transection (SNT). Animal experiments by Klass et al. (2007) and Usach et al. (2017) exhibited that intravenous injection of BMMCs could relieve NP caused by sciatic nerve crush or constriction. In addition to suppressing microglial migration and inflammation associated with nerve injury, a range of angiogenic ligands (basic fibroblast growth factor, angiopoietin-1, VEGF) and cytokines (IL-1b, TNF-a) were secreted by BMMCs to promote local neovascularization and tissue repair (Naruse et al., 2011). BMMCs have unique advantages over bone marrow-derived mesenchymal stem cells in that they are readily available, require no *in vitro* expansion, facilitate transplantation, and minimize the risk of contamination (Alvarez-Viejo et al., 2013).

### 2.5. GABAergic cells

γ-aminobutyric acid (GABA) is an important neurotransmitter that inhibits neurotransmission. The nerve cells in the dorsal horn of the spinal cord that can synthesize GABA are referred to as GABAergic neurons. Upon nerve damage, peripheral or central, GABAergic inhibition at the spinal cord level is decreased, and the excitability of neurons is increased in the dorsal horn of the spinal cord, resulting in the development of persistent post-injury NP (Dugan et al., 2020). Therefore, early intervention aimed to restore GABA levels is an efficacious way to preempt NP development. However, the drugs targeting the GABAergic system (e.g., benzodiazepines, gabapentin, etc.) are of limited value and have side effects (Mason et al., 2018).

Transplantation of GABAergic precursor/progenitor cells to enhance central inhibition and reduce pain is a new approach to managing chronic NP. Hwang et al. (2016) intrathecally transplanted embryonic stem cell-derived spinal GABAergic neural precursor cells into the injury area of the spinal cord. They found that the NP was significantly relieved and the cells survived for more than 7 weeks after transplantation. Manion et al. (2020) using pluripotent stem cell-derived GABAergic interneurons transplantation, also confirmed that the GABAergic cells are involved in spinal cord injury-induced NPs, and synaptic integration occurred after the transplantation. A new analysis reviewed 13 studies on the transplantation of GABAergic precursor cells (Askarian-Amiri et al., 2022), and concluded that GABAergic cells can ameliorate allodynia and hyperalgesia in the rat model, but the extrapolation of the conclusion to mice and other large animals and human subjects still requires a higher-grade evidence. In years to come, multi-specie modeling and multi-model studies on the effect of GABAergic cells on NP are warranted to verify their therapeutic effects.

### 2.6. Genetically-modified cells

While cell transplantation can provide better pain relief than drug therapy, researchers have begun to integrate genetic engineering into stem cell transplantation to improve its efficacy. The neurotrophic factor VEGF introduced into gene-modified neural stem cells can enhance the analgesic effect of NSCs by increasing the expression of VEGF, inducing high-speed migration of NSCs and promoting cell viability (Lee et al., 2016). By transfecting mesenchymal stem cells with fibroblast growth factor (FGF-1; Forouzanfar et al., 2018), glial neurotrophic factor (GDNF; Yu et al., 2015) and sirtuin 1 (SIRT1; Tian et al., 2021) genes, researchers found that the analgesic effect induced by transfected MSCs was better than that of simple mesenchymal stem cells alone. To our knowledge about the mechanism of NP, analgesic peptides, such as inhibitory neurotransmitters (e.g., beta-endorphins), anti-inflammatory peptides (e.g., IL-10), neurotrophic factors (e.g., NT-3, VEGF) and soluble receptors (such as soluble tumor necrosis factor receptor) have potential to serve as targets for genetic engineering (Wang et al., 2020). It is worth mentioning that attention should be paid to the biosafety and the regulation of the gene engineering to avoid the occurrence of occasional tumor-like aggregation caused by overexpression or proliferation of transplanted cells and other possible risks.

## 3. Mechanisms underlying the analgesic effect of cell transplantation

**Figure 1:**
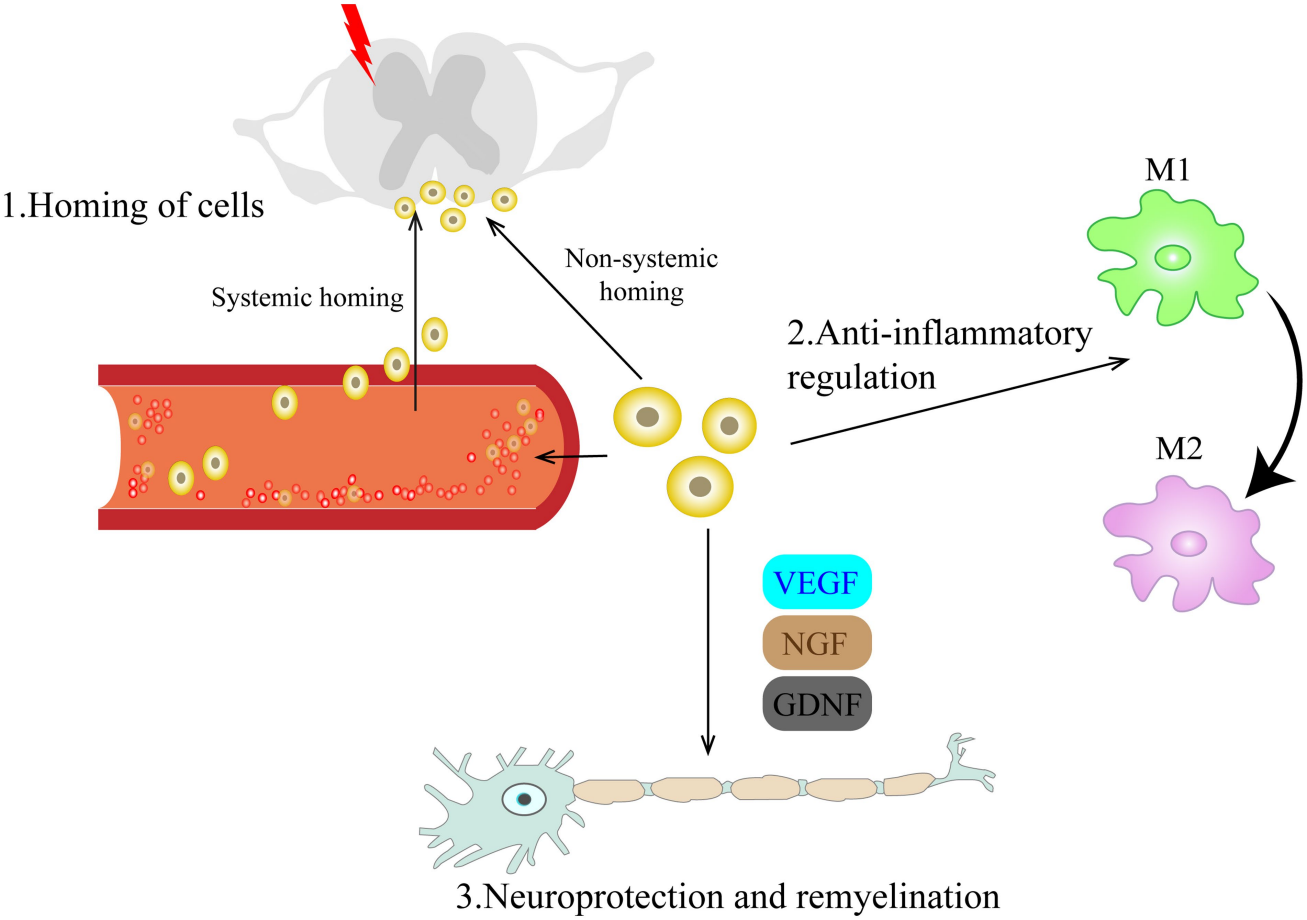
Schematic diagram of the mechanism by which cell therapy promotes recovery from neuropathic pain. The mechanisms involved can be classified as follows: (1) Homing of cells; Cells injected intravenously or intrathecally can migrate directionally to the damaged site under chemokine guidance and provide timely and long-lasting analgesia to NP through a bidirectional interaction with resident cells of the damaged microenvironment in a paracrine manner. (2) Anti-inflammatory regulation; Transplanted cells can modulate microglia activation, inhibit the pro-inflammatory phenotype (M1) of microglia, and promote their polarization to an anti-inflammatory phenotype (M2). (3) Neuroprotection and remyelination. By secreting various trophic regulators, they can promote angiogenesis, axonal regeneration and myelination. VEGF, vascular endothelial growth factor; NGF, nerve growth factor; GDNF, glial cell line-derived neurotrophic factor.

After peripheral or central nerve injury, immune cells accumulate and release glial and pro-inflammatory mediators (nutritional factors, cytokines, and immune transmitters), as a consequence, increasing pain sensitivity, leading to peripheral and central sensitization and inducing NP. Peripheral sensitization is caused by increased cellular excitability resulting from altered expression of various ion channels. Sodium channels (e.g., Nav1.7, Nav1.8, and Nav1.9), transient receptor potential ion channels (e.g., TRPA1, TRPV1) and voltage-gated calcium channels (e.g., Ca_v_3.2, Ca_v_ α_2_ δ_1_) are among common ion channels (Berta et al., 2017; Fernandes et al., 2018; Finnerup et al., 2021). Central sensitization is triggered by glial and astrocyte activation and changes in neuroplasticity in descending inhibitory system (Hosseini et al., 2015). Looking into the above post-injury alternations, multiple studies found that transplanted cells could migrate towards the nerve damage areas induced by chemokines, inhibit neuroinflammation, protect nerves *via* a variety of regulatory mechanisms and promote axonal remyelination (Figure 1).

### 3.1. Homing of transplanted cells

Nerve injury can cause morphological, functional, and behavioral changes at the site of nerve damage, resulting in intractable NP. Hwang et al. (2016) found that intrathecally administered GABAergic cells successfully integrated into or around the injured tissue, with analgesic effect persisting up to 7 weeks post-modeling. Wu et al. (2020) marked the transplanted BM-MSCs with GFP and showed that the cells successfully migrated to the injured segment of spinal cord. In addition to intrathecal injection, intravenously transplanted cells (Siniscalco et al., 2011; Li et al., 2017; Usach et al., 2017) also showed directional migration to the damaged site. Chen et al. (2015) found that BM-MSCs that migrated to the border of the dorsal root ganglia survived for more than 2 months, providing sustained pain relief in mice model induced by sciatic nerve contraction. The process by which transplanted cells migrate to the site of injury is dubbed homing of cells. This phenomenon is beneficial to the survival of transplanted cells, and the migrated cells provide timely and long-lasting analgesia for pain relief by interacting bidirectionally with the residing cells in a damaged microenvironment *via* paracrine secretion (Naruse et al., 2011; Franchi et al., 2014; Du et al., 2019; Kotb et al., 2021).

The homing behavior of transplanted cells has been well described in many studies, but the definitive mechanisms and regulatory processes of their migration process remain unclear (Deak et al., 2010; Liesveld et al., 2020). According to the different transplantation pathways, the homing phenomenon can be divided into systemic homing and non-systemic homing. The directional migration behavior of transplanted cells in the damaged tissue locally or in the adjacent area (e.g., within the sheath) is non-systemic homing. Systemic homing involves multiple steps, including entry of graft cells into circulation, extravasation around the lesion, and interstitial migration toward the injury site (Nitzsche et al., 2017). Although the two processes are different, new advances revealed that the homing of transplanted cells both require chemokine guidance (Siniscalco et al., 2011; Nitzsche et al., 2017; Asgharzade et al., 2020). However, how chemokine receptors such as CCR2 and CXCR4 promote the process of transendothelial extravasation of transplanted cells is not clear, and further clarification of this process will greatly promote the efficiency of homing and overcome one of the existing impediments of cell therapy.

### 3.2. Anti-inflammatory regulation

Activation of microglia and astrocytes leads to neuroinflammation, which plays a pivotal role in the development and persistence of NP (Zhao et al., 2017; Li et al., 2019). Activated glial cells release inflammatory cytokines, leading to upregulation of glutamate receptors. Glutamate released from A-delta and C-fibers is the main nociceptive excitatory neurotransmitter, and is highly associated with the central sensitization of dorsal horn neurons and the maintenance of hyperexcitability (Siniscalco et al., 2007). Transplanted bone marrow-derived mesenchymal stem cells can modulate microglial activation (Forouzanfar et al., 2018; Joshi et al., 2021). Zhong et al. (2020) found that bone marrow MSCs suppressed the M1 phenotype of microglia by secreting GDNF while promoting their conversion to the M2 phenotype, and that the PI3K/AKT signaling pathway is activated during this process.

**Figure 2:**
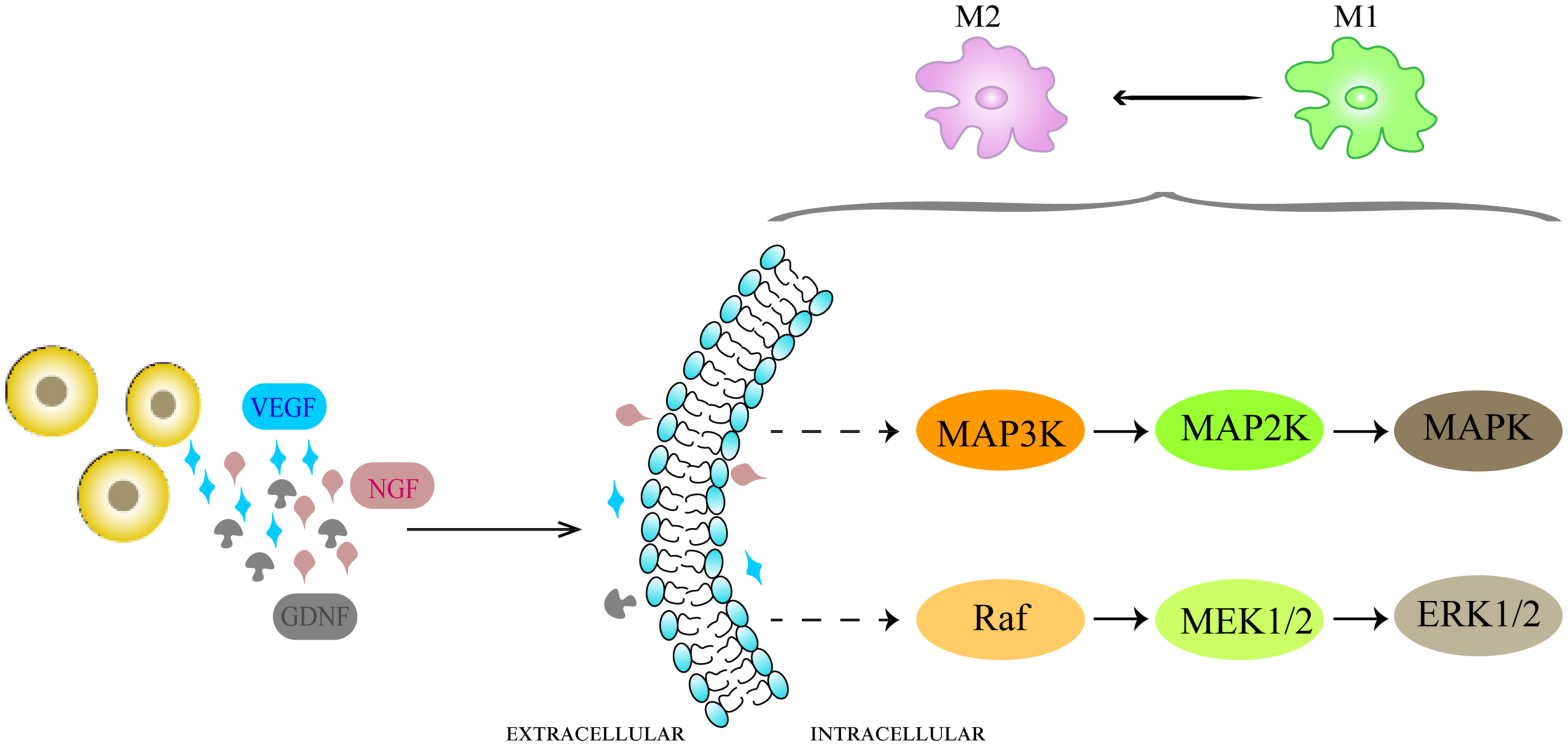
Schematic representation of the mechanisms of anti-inflammatory effects of cell therapy in NP. Neuroinflammation caused by activation of microglia takes part in the development and maintenance of NP. The transplanted cells promote glial cell polarization toward an anti-inflammatory phenotype by inhibiting MAPK-related pathways. The p38 MAPK pathways and ERK 1/2 channels play a central role in neuroinflammation and pain regulation.

Although the mechanism that promotes polarization of glial cells towards an anti-inflammatory phenotype is not fully understood, the researchers found that it is related to the inhibition of MAPK-related pathways (Figure 2). The mammalian mitogen-activated protein kinase (MAPKs) family includes three related pathways, that is, p38 MAPK, extracellular signal-regulated kinase (ERK) and c-Jun N2-terminal kinase (JNK) pathways, and the MAPK cascade is a key signaling pathway governing a wide array of cellular processes, including proliferation, migration, differentiation, apoptosis and responses of cells (Kim and Choi, 2010, 2015). Existing studies have shown that the p38 MAPK pathway and ERK 1/2 channels play a central role in neuroinflammation and pain regulation. Intraspinal injection of BM-MSCs after SCI reportedly inhibited the NF-κ B and p38 MAPK pathways (Zhang et al., 2021). Watanabe et al. (2015) documented that transplantation of MSCs derived from bone marrow inhibited MAPK signaling cascade and inflammatory cell recruitment after spinal cord injury, leading to pain relief. Xie et al. (2019) also found that intrathecally injected BM-MSCs inhibited ERK1/2 up-regulation in the dorsal root ganglia of CCI model rats. However, the exact mechanism by which transplanted stem cells, glial cells and MAPK signaling pathway interact with each other needs further studies at the molecular level.

### 3.3. Neuroprotection and remyelination

Initially, cell transplantation for NP treatment was meant to substitute injured nerve cells, but several studies have shown that the transplanted cells support and enhance the regeneration and remyelination by secreting various trophic regulators of nerve regeneration, such as VEGF, GDNF and NGF, rather than directly replace the lost or damaged cells. Andrews et al. (2016) and Zhang et al. (2019a, 2020a) found that transplanted OECs not only inhibited formation of colloidal scars and cavities after nerve injury, but also promoted regeneration of new axons and remyelination. Lee et al. (2016) found that VEGF-expressing neural stem cells could enhance cell viability under hypoxic conditions and facilitate remyelination of injured sciatic nerves. Transplanted bone marrow mononuclear cells could migrate to the damaged site and promote neovascularization and tissue repair, electromyogram compound muscle action potential (CMPA) verified the presence of regenerated axons (Usach et al., 2017).

## 4. Discussion

Neuropathic pain represents a heterogeneous group of disorders with unfavorable clinical outcomes, and developing an effective and long-lasting analgesic modality has been a challenge for clinicians. Cell therapy is an entirely new approach for addressing NP with promising prospects. At present, multiple animal studies have consistently demonstrated the great potential of cell therapy in the treatment of intractable NP. Cells from a variety of sources have been shown to efficaciously relieve NP, but efficacy and limitations vary with different grafts (Table 1). Apart from murine-derived cell grafts, some researchers also experimentally employed human-derived cells in animal models, and found that they could effectively ease NP (Yousefifard et al., 2016; Manion et al., 2020; Tian et al., 2021; Miyano et al., 2022). Although studies are now principally conducted in animal models of NP, some encouraging clinical findings have been obtained (Feron et al., 2005; Tabakow et al., 2013; Andrews et al., 2016; Curtis et al., 2018; Levi et al., 2018; Zhang et al., 2021). Despite the small number of current clinical studies and the lack of systematic evidence, cell therapy, as a treatment alternative for NP, is undoubtedly worth further exploration.

**Table 1:** Advantages and limitations of different types of cell grafts for the treatment of neuropathic pain models.

Types of cell grafts	Advantages	Limitations
Neural stem cells (NSCs)	Extensive self-regeneration capacity	Low transplant efficiency; malignant transformation possible
Olfactory ensheathing cells (OECs)	Easily available; high cell viability *in vitro*; promote axonal regeneration and myelination	Limited analgesic effect, even causing hyperalgesia
Mesenchymal stem cell (MSCs)	Abundant sources; low immunogenicity; Exosomes: a cell-free approach to transplantation	Gender dimorphism; analgesic effects difference: allodynia superior to hyperalgesia
Bone marrow mononuclear cells (BMMCs)	No need for *in vitro* expansion; facilitate blood vessel formation and tissue repair	May cause neuroapoptosis
GABAergic cells	High survival rate and long survival time	Validate only in rat animal models
Genetically modified cells	Enhance cell viability; improve analgesic effect	Biosafety issues to be considered; risk of tumor formation

Though the current research results are exciting, further research is needed to solve some critical issues, such as optimal transplantation route, transplantation timing, number of transplanted cells, transplantation survival rate, among others.

Cell transplantation can be achieved by a variety of routes, including local injection at the injury area, intravenous, and intrathecal administration. Direct injection into the wounded area saves the migration process after cell transplantation, but may cause needle tract injury, especially NP caused by central nervous system injury and should be used with more caution. Intrathecal transplantation is supposed to be more effective than intravenous transplantation in attaining pain relief and promoting functional recovery by providing sufficient number of cells, but some studies failed to find any statistical difference between the intravenous and intrathecal routes in the improvement of nociceptive hyperalgesia (Yamazaki et al., 2021). Less invasiveness and convenient administration are two advantages of the intravenous route. Although there is a tendency for the transplanted cells to homing to the damaged nerve site, there have been reports that intravenous transplanted cells might be trapped in the lungs, which can cause damage to the transplanted cells and is not conducive to the effect of NP relief (Liu et al., 2017). Intrathecal injection is less damaging than direct targeted injection at the injury site, and the limited intrathecal space and the protection offered by the blood-spinal cord barrier permit a smaller amount of cell fluid to arrive at an effective therapeutic concentration for NP after the intrathecal transplantation (Jwa et al., 2020). With NP due to nerve injury, different grafting modalities may be used for accomplishing optimal effect by taking onto account the differences in the structures surrounding peripheral nerves and central nerves.

With animal models, transplantation roughly falls into acute, subacute, and chronic phases in terms of the timing of transplantation after nerve injury. Watanabe et al. (2015) performed transplantation of BM-MSCs on day 1, 3, 7, and 14 after SCI injury to know whether the timing of transplantation exerted any impact on the improvement of hyperalgesia. They found that transplantation in the acute phase of SCI (especially on the third day after damage) had more favorable result with NP. However, some studies also found that the acute phase of spinal cord injury was not suitable for the survival and differentiation of transplanted BM-MSC due to severe immune inflammatory response, while the scar tissue or cavity formed in the chronic phase was not conducive to axonal growth (Wu et al., 2020). Therefore, 1–2 weeks after nerve injury may be the best time for transplantation. A recent finding was that intravenous transplantation of BM-MSCs 4 days before CCI rat modeling also ameliorated NP (Kotb et al., 2021). This finding may have new implication for the clinical treatment of NP. With the cell therapy for NP, selection of the appropriate timing of transplantation warrants further studies and, over a long time, remains a key issue that has to be addressed for its widespread clinical application.

The optimal volume of transplanted cells is unknown, likely ranging from 1 × 10^5^ to 6 × 10^6^ cell dose/kg with different animal models. A systemic review of MSC transplantation (Wang et al., 2020) found that the number of transplanted cells was seemingly related to animal models and routes of administration. While more studies found that there existed a dose-dependency between the degree of pain relief from cell therapy and the amount of engrafted cells irrespective of central nerve injury or peripheral nerve injury, with a median number of transplanted cells being 3 × 10^6^ cell dose/kg (Hosseini et al., 2015; Han et al., 2019; Askarian-Amiri et al., 2022).

In addition to the transplantation route, transplantation timing and the number of cells, improving the survival rate of transplanted cells is an issue that deserves the attention of cell therapy researchers. Single-cell transplantation has the problems of short cell survival time and low survival rate after transplantation (Franchi et al., 2014). Combination transplantation may be an effective approach. Co-transplantation of NSCs and OECs could promote the survival and proliferation of NSCs, and was beneficial to reverse hyperalgesia (Luo et al., 2013). Gene target modification could also enhance cell viability and improve analgesic effect (Yu et al., 2015; Forouzanfar et al., 2018; Tian et al., 2021), but existing experimental neuralgia models have reported the risk of tumor formation (Yu et al., 2015; Lee et al., 2016). Therefore, more experimentation is needed to regulate the application of genetically modified cells in NP to ensure the biosafety of cell therapy.

Approaches such as scaffolds, microcapsules, and cell sheets have been shown to be beneficial in improving the survival ratio and survival time of transplanted cells. Hsu et al. (2020) found that the analgesic effect of scaffolded stem cells on SNI-induced NP was superior to that of cell transplantation alone, and scaffolds were effective vehicles for the delivery of exosomes because of their good exosome adsorption and slow-releasing nature. Immune rejection after transplantation is one of the factors culpable for the low cell viability. The use of exosomes from MSCs with low immunogenicity can improve cell survival, but the application of exosomes is limited due to the technological restraints and high cost involved in exosome isolation and purification (D’Agnelli et al., 2020). Microcapsules are translucent lipid biofilms with good lipid biocompatibility, wrapped with transplanted cells. The immune barrier prevents inflammatory factors from attacking transplanted cells, improves the survival rate of transplanted cells, and helps relieve NP and repairs nerve damage. Zhang et al. (2020d) found that microencapsulated neural stem cells could better inhibit the overexpression of P2X receptors after nerve injury to relieve NP. In addition, cultured cell sheets were found to express more trophic factors and the cells migrated more easily into the injured area, thereby promoting pain relief and functional rehabilitation (Yamazaki et al., 2021). Although the aforementioned methods can improve the viability of transplanted cells to varying degrees, the related research is still limited to animal experiments, and so far there are no clinical research reports.

Furthermore, while cell therapy is still being actively studied as a treatment alternative for NP, some issues remain to be addressed. First, the current rodent research results cannot be directly extrapolated to humans because rodents and humans differ in species. Optimally, a NP model in non-human primates would provide a higher level of evidence since they have comparable vascular, sensory, and motor systems to humans, but no studies in non-human primate trials have yet been reported. The next issue concerns the gender dichotomy of cellular therapy for NP that has been mentioned in a few animal trials (Schäfer et al., 2014; Coraggio et al., 2018; Hsu et al., 2020). This phenomenon may limit its future clinical application and needs to be verified in further trials. Therefore, these issues have to be resolved to exploit therapeutic potential of cell therapy for NP.

## 5. Conclusion

By reviewing past studies, we can conclude that cell transplantation therapy is a potentially promising therapeutic option for NP. Mounting pre-clinical and clinical studies have shown the potential of cell transplantation-based therapy for NP, but we still have a great deal to explore further. We hope that this review will inform researchers and physicians and facilitate the translation of cell therapy to the clinic.

## Author contributions

QY: conceptualization, writing-original manuscript, and editing. SS and DY: supervision. DY, SS, QY, and TZ: revise the final version before submission. All authors contributed to the article and approved the submitted versio.

## Conflict of interest

The authors declare that the research was conducted in the absence of any commercial or financial relationships that could be construed as a potential conflict of interest.

## Publisher’s note

All claims expressed in this article are solely those of the authors and do not necessarily represent those of their affiliated organizations, or those of the publisher, the editors and the reviewers. Any product that may be evaluated in this article, or claim that may be made by its manufacturer, is not guaranteed or endorsed by the publisher.
